# Prediction models for the risk of gestational diabetes: a systematic review

**DOI:** 10.1186/s41512-016-0005-7

**Published:** 2017-02-08

**Authors:** Marije Lamain – de Ruiter, Anneke Kwee, Christiana A. Naaktgeboren, Arie Franx, Karel G. M. Moons, Maria P. H. Koster

**Affiliations:** 1 Birth Centre, Division Woman and Baby, University Medical Centre Utrecht, KE.04.123.1, PO BOX 85090, 3508 AB Utrecht, The Netherlands; 2 Julius Centre for Health Sciences and Primary Care, University Medical Centre Utrecht, Str. 6.131, PO BOX 85500, 3508 AB Utrecht, The Netherlands; 3 Department of Obstetrics and Gynaecology, Erasmus MC, University Medical Centre, PO Box 2040, 3000 CA Rotterdam, The Netherlands

**Keywords:** First trimester, Gestational diabetes, Model, Prediction, Quality assessment, Systematic review, Validation

## Abstract

**Background:**

Numerous prediction models for gestational diabetes mellitus (GDM) have been developed, but their methodological quality is unknown. The objective is to systematically review all studies describing first-trimester prediction models for GDM and to assess their methodological quality.

**Methods:**

MEDLINE and EMBASE were searched until December 2014. Key words for GDM, first trimester of pregnancy, and prediction modeling studies were combined. Prediction models for GDM performed up to 14 weeks of gestation that only include routinely measured predictors were eligible.

Data was extracted by the CHecklist for critical Appraisal and data extraction for systematic Reviews of prediction Modelling Studies (CHARMS). Data on risk predictors and performance measures were also extracted. Each study was scored for risk of bias.

**Results:**

Our search yielded 7761 articles, of which 17 were eligible for review (14 development studies and 3 external validation studies). The definition and prevalence of GDM varied widely across studies. Maternal age and body mass index were the most common predictors. Discrimination was acceptable for all studies. Calibration was reported for four studies. Risk of bias for participant selection, predictor assessment, and outcome assessment was low in general. Moderate to high risk of bias was seen for the number of events, attrition, and analysis.

**Conclusions:**

Most studies showed moderate to low methodological quality, and few prediction models for GDM have been externally validated. External validation is recommended to enhance generalizability and assess their true value in clinical practice.

**Electronic supplementary material:**

The online version of this article (doi:10.1186/s41512-016-0005-7) contains supplementary material, which is available to authorized users.

## Background

Gestational diabetes mellitus (GDM), diabetes diagnosed by oral glucose tolerance test (OGTT) in the second or third trimester that is not clearly overt diabetes [1], is becoming the number one complication in pregnancy. Over the past decade, the prevalence of GDM has rapidly risen and ranges from 3 up to 35% [2, 3] depending on the definitions used and populations studied [4, 5]. This parallels the emerging trends in obesity, population aging, and diabetes mellitus type II. The rising prevalence of GDM contributes to an increasing number of adverse perinatal outcomes, such as macrosomia, shoulder dystocia, caesarean delivery, and neonatal hypoglycemia [6]. Moreover, GDM has a major impact on long-term maternal health as well as neonatal health. The mother is at high risk to develop diabetes mellitus type II within 5 years after pregnancy [7–9], and her child is at increased risk of developing childhood obesity and metabolic syndrome [10–12]. Early diagnosis of GDM will allow for timely treatment, such as dietary counseling or pharmacotherapy, which has been shown to be effective for the improvement of perinatal outcomes [13–15].

Early risk stratification by prediction modeling might offer opportunities to improve care for those women at high risk of developing GDM. As timely intervention is the key to preventing (or reducing) adverse outcomes in GDM, clinicians need prediction models that can be used in the first trimester. Additionally, as all pregnancies should be assessed for the risk of developing GDM, models that only require easily obtained information are preferable. Although various prediction models for GDM have been developed, they are not widely used in routine clinical practice. Ideally, new prediction models are externally validated and updated before they are implemented. A systematic review describing the characteristics of the model development, the included predictors, outcome measurement, and whether they have undergone external validation will provide insight into the current quality of first-trimester GDM prediction models. This will improve validation and implementation of prediction models for GDM. For this purpose, we generated a comprehensive overview of all published first-trimester prediction models for GDM consisting of routinely measured predictors and assessed the methodological quality of these studies.

## Methods

The specifics of our research question, which was framed according to the CHecklist for critical Appraisal and data extraction for systematic Reviews of prediction Modelling Studies (CHARMS) guidance [16], are shown in Table 1. The results have been reported conforming to the PRISMA statement [see Additional file 1].

**Table 1:** Framework of the research question

Item	Description
Intended scope of the review	Reviewing prognostic models that are aimed at predicting the development of gestational diabetes in pregnancy
Type of prediction modeling studies	Both model development and model validation studies
Target population to whom the prediction model applies	Low- and medium-risk pregnant women in the first trimester of pregnancy
Outcome to be predicted	Probability of developing gestational diabetes in current pregnancy
Intended moment of using the model	First trimester of pregnancy

### Search strategy

We performed a computerized systematic search in MEDLINE and EMBASE on December 17, 2014. Key words for GDM and first trimester of pregnancy were combined with a validated search strategy for prediction modeling studies [17]. Detailed information on the exact search syntax is presented in Additional file 2. Reference lists of the selected articles were scanned to ascertain that no relevant articles were missed.

### Study selection

In this systematic review, we aimed to identify all published prognostic prediction models that are applicable in the first trimester of pregnancy (up to 14 weeks of gestational age). Moreover, we focused on reviewing the prediction models including routinely measured predictors only (i.e., predictors based on maternal characteristics, anthropometric measures, or glucose measurement) to enhance the generalizability of our review.

Model development studies as well as validation studies were eligible. Eligibility assessment of studies was independently performed by two authors (MLdR, MPHK) by screening the title and abstract. Exclusion criteria for selection were preconception prediction, univariate prediction studies, diagnostic models, prediction models including invasive measures (e.g., biomarkers, ultrasound measures), association studies of one or more variables and the outcome, no primary reports (e.g., systematic reviews), conference abstracts, and other languages than English, French, or German. Next, full-text articles were thoroughly reviewed by two authors (MLdR, MPHK). Any disagreement between reviewers was resolved by consensus. Authors of the original studies were contacted by e-mail for further information if necessary.

### Assessment of methodological quality

For critical appraisal of the individual studies, we used the recently published CHARMS checklist [16]. In summary, the following items of the CHARMS checklist were handled: study characteristics and participants; outcome to be predicted; candidate predictors (for development studies only); sample size and handling of missing data; model development (for development studies only); model evaluation; and results and interpretation.

One reviewer (MLdR) extracted data according to the CHARMS checklist from the included studies. A second author (MPHK) checked the extracted data. Disagreements were resolved by consensus between these two authors. After data extraction, each study was scored for risk of bias as follows: “low” if bias was unlikely; “moderate” if there were no essential shortcomings, but not all criteria were satisfied; and “high” if bias was very likely due to essential errors in one or more of the domains [18].

### Data extraction

For each included study, the method of GDM diagnostic strategy and criteria were obtained in detail. Moreover, risk predictors that were included in the prediction model as well as indicators of performance measures were extracted. The actual predictive performance was also extracted and reported as the area under the (receiver operating) curve (AUC or *c*-statistic) or as classifications measures, such as sensitivity, specificity, positive predictive value, and negative predictive value.

## Results

### Study selection

**Fig. 1:**
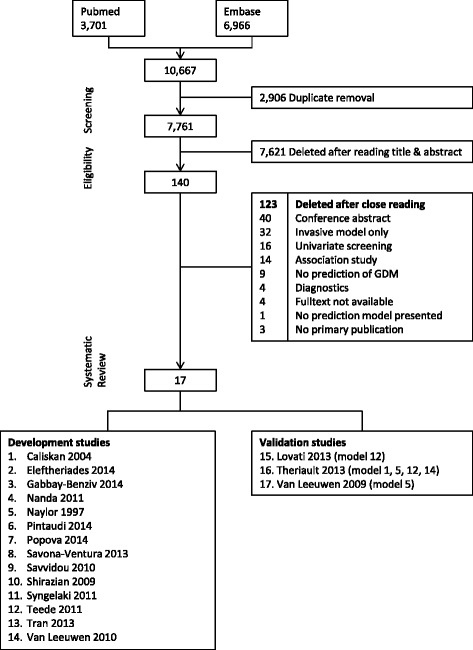
Flow chart of systematic review. Flow chart of systematic review of first-trimester prediction models for gestational diabetes

An overview of the flow of the systematic review process is given in Fig. 1. Our computerized search yielded a total of 7761 unique articles. Of these, 7621 articles were excluded on the basis of the title and abstract and another 123 articles on the basis of full-text screening. Reference cross-checking of the selected papers yielded no additional studies. Thus, a total of 17 studies on first-trimester prediction modeling for GDM were identified for inclusion in this review [19–34]. Fourteen studies were development studies and another three studies were external validation studies. All studies were published between 1997 and 2014 and conducted in 11 different countries: three in the UK, two in Italy, two in the Netherlands, two in Greece, two in Canada, and one in Australia, Iran, Russia, Turkey, the USA, or Vietnam.

### Outcome assessment

All extracted data on diagnostic strategies for GDM are shown in Additional file 3. Eight different diagnostic outcome (i.e., GDM) criteria were used in the included studies. The prevalence of GDM within the included studies ranged from 2.4% (model 14) to 26.5% (model 7). The diagnostic criteria differed in the dose of oral glucose to be administered, number of glucose levels measured, time interval of glucose measures, and cutoff point of glucose levels. The oldest criteria used, by Carpenter and Coustan and the National Diabetes Data Group, were the only criteria that prescribed a 100-g dose of glucose and a four-point blood glucose level measurement. All other, more recent, GDM criteria used a 75-g dose of glucose.

The included studies had different strategies for setting the diagnosis of GDM: six studies used a one-step approach by applying an OGTT to all participants for diagnosing GDM (models 2, 6, 8, 10, 13, and 15). In another five studies, a two-step approach was performed using a 50-g glucose challenge test followed by an OGTT (models 1, 3, 5, 12, and 16). Five studies used another two-step approach, in which a screening method (i.e., random glucose, risk factor, or a combination of screening) was followed by an OGTT (models 4, 9, 11, 14, and 17). The remaining study did not clearly report their strategy, but they did report on their diagnostic criteria (model 7).

### Risk of bias assessment

**Fig. 2:**
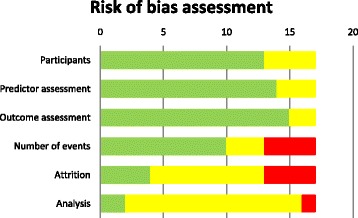
Risk of bias assessment summary. Risk of bias assessment for six predefined domains for each included study. Legend: *green*, low risk of bias; *yellow*, medium risk of bias; *red*, high risk of bias

Table 2 shows the risk of bias for each included study based on six predefined domains, and Fig. 2 provides a summarized overview of potential bias. For participant selection, predictor assessment, and outcome assessment, the majority of the studies were scored as low risk of bias (*n* = 13–15; 76–88%). None of the studies had a high risk of bias in these categories. A moderate risk of bias for participant selection was mainly due to debatable inclusion or exclusion criteria. Predictor assessment was at moderate risk for three models because assessment of predictors was performed in retrospect, after the outcome (GDM) was known. Two studies had a moderate risk of bias regarding outcome assessment due to different reasons: (1) two different diagnostic criteria for GDM were used in the study period (model 15) and (2) diagnosis of GDM based on risk factor screening only, which is a less sensitive approach (model 9) [35].

**Table 2:** Risk of bias assessment

Study	Participants	Predictor	Outcome	No. of events	Attrition	Analysis
1. Caliskan 2004	L	L	L	L	M	H
2. Eleftheriades 2014	M	L	L	M	L	M
3. Gabbay-Benziv 2014	L	L	L	H	M	M
4. Nanda 2011	L	L	L	L	H	M
5. Naylor 1997	L	L	L	M	M	M
6. Pintaudi 2014	L	M	L	L	M	M
7. Popova 2014	L	M	L	L	H	M
8. Savona-Ventura 2013	M	M	L	L	M	M
9. Savvidou 2010	L	L	M	L	H	M
10. Shirazian 2009	L	L	L	H	M	M
11. Syngelaki 2011	L	L	L	H	M	M
12. Teede 2011	L	L	L	L	H	M
13. Tran 2013	L	L	L	L	M	M
14. Van Leeuwen 2010	M	L	L	H	L	L
15. Lovati 2013	M	L	M	L	L	M
16. Theriault 2014	L	L	L	L	M	M
17. Van Leeuwen 2009	L	L	L	M	L	L

*Abbreviations*: *L* low risk of bias, *M* medium risk of bias, *H* high risk of bias

The number of events was scored as high risk of bias for four models (24%) because they had less than six events per variable (EPV) or because we were unable to extract the EPV. A moderate risk was scored for three (18%) other prediction models with an EPV between six and ten or with a very low number of cases (<50) for external validation.

Assessment of attrition (i.e., no loss to follow-up) showed a high risk of bias for four (24%) of the prediction models. These four studies were scored as high risk due to lack of information on sample flow or on missing data. Most studies (*n* = 9, 53%) performed a complete case analysis; these models were scored as moderate risk of bias.

Information on development of the prediction models was insufficiently reported in almost all studies (*n* = 14; 82%), and therefore, all scored a moderate risk. Only two studies (from the same research group) reported a complete description of the analysis performed (models 14 and 17). A high risk of bias was present in one study where no information on model development was provided (model 1).

### Predictors in the final model

An overview of the predictors in the final models in each study is shown in Table 3. The smallest final prediction model consisted of two predictors and the largest of eight predictors. Age and body mass index were the most common predictors; both were included in 11 prediction models. Four models included other anthropometric measures, i.e., maternal weight, blood pressure, and abdominal circumference. Risk factors based on obstetric history were often included; five models included a history of GDM and four a history of macrosomia. Five models included a positive family history of diabetes. Routine obstetric care often includes a blood glucose level measurement at the beginning of pregnancy to rule out pre-existing diabetes. Three models included this glucose level measurement.

**Table 3:** Calibration and discrimination of development studies

Study	Risk predictors		AUC			Calibration	Sensitivity	Specificity
	Predictors and no. of predictors		Original study	External validation 1	External validation 2			
1. Caliskan 2004	Poor outcome, age, BMI, fam hx of DM, hx of macrosomia	5	NR	0.68 [0.65–0.71]			85.7 [67.4–100]	67 [59.4–74.8]
2. Eleftheriades 2014	Weight, age	2	0.73 [0.65–0.81]				32.4 NR	90 NR
3. Gabbay-Benziv 2014	Age, ethnicity, hx of GDM, hx of macrosomia	5	0.81 [0.77–0.87]			*p* = 0.18	85 [74.6–93.2]	62 [57.4–64.0]
4. Nanda 2011	Age, BMI, ethnicity, hx of GDM, hx of macrosomia	5	0.79 [0.76–0.82]				52.9 NR	90 NR
5. Naylor 1997	Age, BMI, ethnicity	3	0.69 NR	0.67 [0.64–0.70]	0.64 [0.56–0.72]	*p* = 0.93	65.9 NR	84 NR
6. Pintaudi 2014	BMI, glucose, hx of macrosomia, fam hx of DM	4	NR				89 NR	40 NR
7. Popova 2014	BMI, glucose, AC, PCOS	4	NR				NR	NR
8. Savona-Ventura 2013	Age, glucose, blood pressure	3	0.89 [0.86–0.91]				96.6 NR	37.5 NR
9. Savvidou 2010	Age, BMI, ethnicity, hx of GDM, mode of conception, parity, smoking	7	0.82 NR				NR	NR
10. Shirazian 2009	Age, BMI, fam hx of DM	3	NR				NR	NR
11. Syngelaki 2011	Age, BMI, ethnicity, mode of conception, smoking, hx of chronic hypertension, parity, hx of macrosomia	8	NR				NR	NR
12. Teede 2011	Age, BMI, ethnicity, fam hx of DM, hx of GDM	5	0.70 NR	0.74 [0.70–0.78]	0.60 [0.56–0.64]		68.0 [61.3–73.9]	70.8 [68.8–72.6]
13. Tran 2013 ADA criteria	Age, BMI	2	0.71 [0.68–0.75]				79.9 NR	48.5 NR
13. Tran 2013 IADPSG criteria	Age, BMI, fam hx of DM	3	0.65 [0.62–0.67]				70.4 NR	52.5 NR
13. Tran 2013 WHO criteria	Age, BMI	2	0.63 [0.60–0.65]				65.1 NR	53.7 NR
13. Tran 2013 ADIPS criteria	Age, BMI	2	0.64 [0.62–0.67]				64.1 NR	56.8 NR
14. Van Leeuwen 2010	BMI, ethnicity, fam hx of DM, hx of GDM	5	0.77 [0.69–0.85]	0.76 [0.73–0.79]		*p* = 0.25	45.8 [28.2–64.5]	88.4 [87.9–88.8]

*Abbreviations*: *AC* abdominal circumference, *AUC* area under the (receiver operating) curve, *BMI* body mass index, *DM* diabetes mellitus, *fam* family, *GDM* gestational diabetes, *hx* history, *NR* not reported, *PCOS* polycystic ovary syndrome

### Predictive performance

Table 3 summarizes the predictive performance of the prediction models. The *c*-statistic of nine of the development studies that reported predictive performance ranged from 0.63 to 0.89. The three external validation studies showed *c*-statistics from 0.60 to 0.76. Median sensitivity and specificity were 67 and 71% and 66 and 65% for development and validation studies, respectively.

Although external validation is necessary to assess the true value of prediction models, the majority of developed models (71%) has not yet been externally validated. Two models (models 5 and 12) used an internal validation technique, and four of the developed models were externally validated (models 1, 5, 12, and 14). Their external performance measures were slightly lower compared to the original results.

Calibration was reported for four studies (24%; models 3, 5, 14, and 17), by means of a Hosmer-Lemeshow test, a *χ* ^2^ goodness of fit, or a calibration plot. The external validation of model 5 showed a poor goodness of fit (*p* = 0.06); the other three models showed adequate calibration.

## Discussion

### Main findings

In this systematic review on first-trimester prediction models for GDM, consisting of routine measures only, we identified 14 development studies and three external validation studies based on four of the developed models. Assessment of methodological quality revealed various shortcomings on the model development studies, resulting in a moderate to low quality of the reviewed models.

These shortcomings all lead to overfitted prediction models. Overfitting means that a prediction model is too closely tailored to the data at hand, which makes it less likely for a model to perform well in practice, in the same or in a different population. The likeliness of overfitting is high, as most authors did not report on the number of candidate predictors they considered or on the predictor selection technique used (e.g., dichotomization of variables, univariable significance criteria for inclusion). Additionally, handling of missing data can also be a source of bias. Only two studies handled missing data according to the most preferable standards, i.e., by multiple imputation [36]. At last, as a crucial step prior to implementation, validation of developed prediction models in external datasets (i.e., datasets that were not used to develop the model) is needed. All development studies described in this review have a high risk of bias, which often show overestimated performance measures. We found that only four out of the 14 identified models have been externally validated, despite knowing that external validation in independent data is all that matters. The models showing the highest *c*-statistic in our review have not yet been externally validated (i.e., models 3, 8, and 9).

### Strengths and limitations

To our knowledge, this is the first systematic review on prediction models for GDM. As the number of prediction models for GDM is rapidly increasing, it is important to generate an overview of the quality and characteristics of models that are already available. A major strength of our review is that it is based on a validated search strategy for prediction models. Furthermore, all prediction models were thoroughly assessed on quality by means of the CHARMS guideline.

However, some limitations need to be addressed. We restricted our inclusion criteria to models consisting of routine measures only. Therefore, promising prediction models that also use more invasive measurements may have been missed. Leaving aside that studies on the added value of biomarkers to noninvasive models are scarce, the biomarkers that have been studied for the prediction of GDM show that their predictive performance is limited and contradictory results have been published [32, 37–39]. For future studies, we recommend to assess the added value of biomarkers only for prediction models that have been proven to perform well in external validation studies.

A second limitation of our systematic review might be the highly variable measures of outcome, which hampers the comparison of prediction models for GDM. GDM was diagnosed by eight different criteria and by even more diagnostic strategies, reflecting the variation in currently used international diagnostic criteria [40, 41]. Though sub-analysis according to the criteria used would be interesting, we expect subgroups to be so small that this will severely limit the value of sub-analysis. Moreover, it is known that the differentiation in diagnostic strategies and criteria has a major impact on the prevalence of GDM [31, 41]. There is an international guideline for diagnostic strategies and criteria for GDM [42], but international implementation is hampered by the ongoing debate on a “gold standard.” For a fair comparison of prediction models for GDM, universally implemented diagnostic strategy and criteria of GDM would be of great benefit.

### Interpretation

Our systematic review identified multiple prediction models for GDM in the first trimester of pregnancy consisting of routine measures only, most of them showing moderate to low methodological quality. Correspondingly, other systematic reviews on prognostic models in different fields (i.e., cancer prognosis, low back pain prognosis, and prognosis of pregnancy complications) also report the frequent occurrence of inadequate methods for development of prediction models [43–45]. The recently published guidelines that advocate for transparent reporting of prediction models may function as a tool to improve reporting on methodological quality, also in obstetric research [46].

Although most studies showed promising predictive performance in development studies, this systematic review shows there is an urgent need of external validation of the most promising ones. A recently published external validation study did not validate the models with the best performance measures [47]. The lack of external validation of these results leads to limited generalizability, as development data often leads to inaccurate predictions when applied to other individuals than the individuals in the original study [16]. Therefore, we strongly advocate an external validation and head-to-head comparison of all models that were identified in this systematic review.

Assuming that performances reported in development studies may be confirmed in external validation studies, prediction models for GDM show a performance at least as good as traditional risk factor screening, as recommended by current guidelines [48, 49]. However, prediction model-based GDM screening might offer the opportunity to reduce the burden of diagnosing GDM (e.g., only applying an OGTT to women at high risk of developing GDM). Current guidelines for GDM diagnostic strategies show a high sensitivity (>90%), but a very low specificity (3–35%), therefore requiring the administration of an OGTT to the majority of the population (up to 97%) [4]. Hopefully, when prediction models will be implemented into routine obstetric care, fewer women undergo an OGTT while still maintaining the high sensitivity. Therefore, a prediction model based on routine measures will probably also be a cost-effective intervention. There will also be opportunities for prevention of GDM as models can be applied as early as the first trimester of pregnancy. Knowledge on the efficacy of prevention of GDM is not yet conclusive as several trials are still ongoing [50]. Preventive strategies and targeted care would be in line with a greater trend in health care towards a more personalized approach of health care delivery: “the right treatment for the right person at the right time” [51].

## Conclusions

Although many first-trimester prediction models for GDM have been developed, only few have been externally validated and most showed moderate to low methodological quality. Before implementation of prediction models in clinical practice can take place, it is important that their true value is assessed by external validation in the population in which they are to be used. As the best and most promising prediction models have not yet been externally validated, we recommend an external validation and head-to-head comparison of these models before including them in clinical guidelines and daily practice. Hopefully, this will guide implementation of prediction models for GDM into clinical practice and provide room for targeted interventions in pregnancy.

## Additional files

Additional file 1: PRISMA 2009 Checklist. The file contains the PRISMA checklist and indicates were data is described in the manuscript. (DOCX 29 kb)

Additional file 2: Search string. The file contains the full search string used for our computerized search of prediction models for gestational diabetes mellitus. (DOCX 14 kb)

Additional file 3: CHARMS checklist for each included study (full version). The file contains all data extracted from the studies included in our systematic review conforming to the CHARMS checklist. (DOCX 47 kb)

## Abbreviations

AUC: Area under the (receiver operating) curve
CHARMS: CHecklist for critical Appraisal and data extraction for systematic Reviews of prediction Modelling Studies
EPV: Events per variable
GDM: Gestational diabetes mellitus
OGTT: Oral glucose tolerance test
