# Supplementary material for: Prediction models for the risk of gestational diabetes: a systematic review
Source: Diagn Progn Res. 2017 Feb 8;1:3. doi: 10.1186/s41512-016-0005-7 (PMC6457144; doi:10.1186/s41512-016-0005-7)
Supplement: Supplementary file 2 — Search string. The file contains the full search string used for our computerized search of prediction models for gestational diabetes mellitus. (DOCX 14 kb) [file 41512_2016_5_MOESM2_ESM.docx]

**Additional file 2. Search string**

Full search syntax for MEDLINE through Pubmed.

**Gestational diabetes**

(GDM[tiab] OR (Gestational[tiab] AND Diabetes[tiab]) OR (Pregnancy[tiab] AND induced[tiab] AND diabetes[tiab]) OR (Pregnancy[tiab] AND related[tiab] AND diabetes[tiab]) OR (gestational[tiab] AND Metabolic[tiab] AND disorder[tiab]) OR (Gestational[tiab] AND glucose[tiab] AND intolerance[tiab]) OR (pregnancy[tiab] AND Hyperglycaemia[tiab]) OR "Diabetes. Gestational"[Mesh]) AND

**First trimester**

(((First[tiab] OR early[tiab] OR initial[tiab] OR inaugural[tiab] OR premier[tiab] OR primal[tiab] OR primary[tiab] OR prime[tiab]) AND (quarter*[tiab] OR trimester*[tiab] OR pregnan*[tiab] OR gestat*[tiab] OR gestosis[tiab] OR gravidit*[tiab])) OR first-trimester[tiab] OR ((9[tiab] OR nine[tiab] OR 10[tiab] OR ten[tiab] OR 11[tiab] OR eleven[tiab] OR 12[tiab] OR twelve[tiab] OR 13[tiab] OR thirteen[tiab] OR 14[tiab] OR fourteen[tiab] OR 15[tiab] OR fifteen[tiab]) AND (week*[tiab] OR pregnan*[tiab])) OR pregnan*[tiab] OR "pregnancy"[MeSH] OR "Pregnancy Trimester. First"[Mesh] OR "Pregnant Women"[Mesh]) AND

**Prediction modeling**

((Validat*[tiab] OR Predict*[ti] OR Rule*[tiab]) OR (Predict*[tiab] AND (Outcome*[tiab] OR Risk*[tiab] OR Model*[tiab])) OR ((History[tiab] OR Variable*[tiab] OR Criteria[tiab] OR Scor*[tiab] OR Characteristic*[tiab] OR Finding*[tiab] OR Factor*[tiab]) AND (Predict*[tiab] OR Model*[tiab] OR Decision*[tiab] OR Identif*[tiab] OR Prognos*[tiab])) OR (Decision*[tiab] AND (Model*[tiab] OR Clinical*[tiab] OR Logistic Model*[tiab])) OR (Prognostic[tiab] AND (History[tiab] OR Variable*[tiab] OR Criteria[tiab] OR Scor*[tiab] OR Characteristic*[tiab] OR Finding*[tiab] OR Factor*[tiab] OR Model*[tiab])) OR ("risk score"[All fields] OR "prediction model"[All fields] OR "prediction rule"[All fields] OR "risk assessment"[All fields] OR "algorithm"[All fields]) OR ("stratification" OR "ROC Curve"[MeSH] OR "discriminate" OR "c-statistic" OR "c statistic" OR "area under the curve" OR "AUC" OR "calibration" OR "indices" OR "algorithm" OR "Multivariable"))

NOT ("animals"[MeSH] NOT "humans"[MeSH])
