# Supplementary material for: Prediction models for the risk of gestational diabetes: a systematic review
Source: Diagn Progn Res. 2017 Feb 8;1:3. doi: 10.1186/s41512-016-0005-7 (PMC6457144; doi:10.1186/s41512-016-0005-7)
Supplement: Supplementary file 3 — CHARMS checklist for each included study (full version). The file contains all data extracted from the studies included in our systematic review conforming to the CHARMS checklist. (DOCX 47 kb) [file 41512_2016_5_MOESM3_ESM.docx]

Additional file 3. CHARMS checklist for each included study (Full version)

| Study | | **1. Caliskan 2004** | **2. Eleftheriades 2014** | **3. Gabbay-Benziv 2014** |
| --- | --- | --- | --- | --- |
| Definitions | |  |  |  |
| Objective | *Primary study objective*  *[Prediction model development (D) or validation (V) study]* | Effectiveness of own risk score for GDM [D & V] | Development of first trimester prediction model for GDM [D] | First trimester prediction of GDM by maternal characteristics [D] |
| Source of data | Source of data (e.g. cohort, case-control, etc) | Prospective cohort | Nested case-control study | Prospective cohort |
| Participants | - *Participant eligibility and recruitment method (e.g. consecutive participants, location, number of centres, in or exclusion criteria)* - *Number of participants* - *Participant description* - *Study dates* | - D: 429 particpants, 1999, nested case-control - V: 422 participants, 2000, single centre, Turkey - recruitment: 24-28 wks - exclusion: hx of DM, multiple gest. - baseline characteristics presented | - 134 participants, dates NR, single centre, Greece - recruitment: 11-14 wks - exclusion: hx of hypertension, hx of GDM, chromosomal abnormalities in fetuses, multiple gest., hx of DM, - baseline characteristics presented | - 948 participants, 2007 – 2010, multi centre, USA - recruitment: 11-14 wks - exclusion: hx of DM, multiple gest. - baseline characteristics presented |
| Outcomes to be predicted | - *Definition and method for measurement of outcome* - *The same definition used in all patients?* - *The outcome assessment without knowledge of candidate predictors?* | - GDM: NDDG - 2-step: 50g GCT (7.2^a^), 24-28 wks - outcome assessment blinded | - GDM: IADPSG - 1-step, 24-28 wks - outcome assessment not blinded | - GDM: Carpenter&Coustan - 2-step: 50g GCT (7.5), 24-28 wks - outcome assessment not blinded |
| Candidate predictors | - *Definition and method for measurement of candidate predictors* - *Timing of predictor assessment* - *Were predictors assessed blinded for outcome?* - *Handling of predictors in the modelling (e.g. continuous, linear, non-linear transformations, etc.)* | - demographics, patient history, physical examination derived from clinical record - assessment blinded for outcome - D: 9 risk factors from literature, 3 dichotomized, 6 dichotomous variables | - demographics, patient history, physical examination recorded in database - assessed at booking, before diagnosis - 3 continuous, 2 dichotomous variables | - questionnaire on demographics, patient history; physical examination - assessed at booking, before diagnosis - selection by linear association with screening test result - 8 continuous, 1 categorical, 5 dichotomous variables |
| Sample size | - *Number of events* - *Number of events in relation to candidate predictors (EPV)* | - 14 GDM cases (3.3%)  - EPV – D: 15.9 | - 40 GDM cases  - EPV 8 | - 63 GDM cases (6.8%) - EPV 4.5 |
| Missing data | - *Number of participants with any missing value* - *Number of participants with missing data for each predictor* - *Handling of missing data (e.g. complete-cases, imputation)* | - missing outcome excluded (0.7%)  - missing predictors: NR | - missing data: NR | - missing outcome excluded (2.5%)  - missing predictors: NR |
| Model development | - *Modelling method* - *Method for selection of predictors for inclusion in multivariable modelling* - *Method for selection of predictors during multivariable modelling (e.g. forward, backward, etc) and criteria used (e.g. p-value, Akaike Information Criterion)* - *Shrinkage of predictor weight or regression coefficients* | - multivariable logistic regression - predictor selection: all candidate predictors, Forward Wald method; criteria: NR - shrinkage: NR | - multivariable logistic regression - predictor selection: backward selection; criteria: NR - shrinkage: NR | - multivariable logistic regression - predictor selection: backward selection; criteria: NR - shrinkage: NR |
| Model performance | - *Calibration and discrimination measures* - *Classification measures* | - calibration: NR, discrimination: NR - classification measures reported for different screening strategies | - calibration: NR, discrimination: AUC - classification measures reported for fixed FPRs | - calibration: Hosmer-Lemeshow - discrimination: AUC |
| Model evaluation | - *Method used for testing model performance: development dataset only or separate external volition* - *In case of poor validation, whether model was adjusted or updated* | - NR | - NR | - NR |
| Results | - *Final and other multivariable models presented, including predictor weights, intercept, baseline survival, model performance* - *Any alternative presentation (e.g. sum score, nomogram)* | - clinical risk score | - regression model with odds ratios - intercept: NR | - full regression model - Excel spreadsheet for clinicians to derive risk score |
| *Interpretation and discussion* | - *Interpretation of presented models* - *Comparison with other studies, discussion, generalizability* | - risk factor scoring decreases unnecessary testing | - effective screening by the combination of maternal factors and PlGF | - model is accurate enough to identify women who will benefit from early screening |

|  | **4. Nanda 2011** | **5. Naylor 1997** | | **6. Pintaudi 2014** | | **7. Popova 2014** | | **8. Savona-Ventura 2013** | |
| --- | --- | --- | --- | --- | --- | --- | --- | --- | --- |
| Objective | Develop model for GDM based on maternal characteristics [D] | Screening for GDM on basis of maternal characteristics [D &V] | | Identify risk factors and high risk subgroups for GDM [D] | | Identify early markers of GDM [D] | | Identify clinical risk factors for GDM among Mediterranean women [D] | |
| Source of data | Prospective cohort | Prospective cohort | | Retrospective cohort | | Retrospective cohort | | Prospective cohort | |
| Participants | - 11.464 participants, 2006 - 2009, single centre, UK - recruitment at booking - inclusion: singleton gest., >30 wks and phenotypically normal infant - exclusion: hx of DM - baseline characteristics presented | - 3.152 patients, 1989 - 1992, multicentre, Canada - recruitment < 24 wks - inclusion: ≥24 yrs, singleton pregnancy - exclusion: hx of DM, delivery <28 wks | | - 1.028 participants, 2010 - 2011, single centre, Italy - recruitment: NR - exclusion: hx of DM - baseline characteristics presented | | - 548 participants, 2011 - 2012, single centre, Russia - recruitment: < 13wks - exclusion: hx of DM, alcohol or drugs, diseases/drugs affecting carbohydrate metabolism - baseline characteristics presented | | - 1368 participants, 2010 - 2011, multi-centre, Mediterranean - recruitment at diagnostic test - exclusion: hx of DM - baseline characteristics presented | |
| Outcomes to be predicted | - GDM: WHO - 2-step: glucose (6.7), 24-28 wks - outcome assessment blinding NR | - GDM: Carpenter&Coustan  - 2-step: 50 g GCT (?), 26 wks  - outcome assessment not blinded | | - GDM: IADPSG - 1-step, 24-28wks - outcome assessment not blinded | | - GDM: IADPSG - screening: NR, 24-28 wks - outcome assessment not blinded | | - GDM: ADA - 1-step, 24-32 wks - outcome assessment not blinded | |
| Candidate predictors | - questionnaire on demographics, patient history; physical examination - 2 continuous, 2 categorical, 3 dichotomous variables - predictor assessment at booking | - demographics, patient history, physical examination recorded - 2 categorized, 2 categorical variable, 2 dichotomous variables - predictor assessment at booking | | - demographics, patient history, physical examination recorded - 3 dichotomized, 4 dichotomous variables - predictor assessment in retrospect | | - demographics, patient history, physical examination recorded - 4 continuous, 6 dichotomous variables - predictor assessment in retrospect | | - demographics, patient history, physical examination at clinic - 7 continuous, 6 dichotomous variables - predictor assessment at diagnosis | |
| Sample size | - 297 GDM cases (2.6%) - EPV 42.4 | - D: 44 GDM (2.8%) - V: 69 GDM (4.4%) - EPV – D: 7.3 | | - 114 GDM cases (11.1%) - EPV 16.3 | | - 145 GDM cases (26.4%) - EPV 14.5 | | - 119 GDM cases (8.7%) - EPV 9.9 | |
| Missing data | - missing outcome excluded (NR %) | - excluded if information not sufficiently complete (0.7%) | | - excluded if predictor missing (1.3%) | | - missing data: NR | | - complete case analysis - missing outcome (8.5%) | |
| Model development | - multivariable logistic regression - predictor selection: backward selection; criteria: NR - shrinkage: NR | - multivariable logistic regression - predictor selection: if p<0.05 in univariate analysis - shrinkage: NR | | - multivariate logistic regression   predictor selection: RECPAM, minimum of 20 cases and 50 women per node – backward with RECPAM classes forced in | | - multivariate logistic regression - predictor selection: Decision Tree analysis | | - multivariable logistic regression - predictor selection: forward stepwise, in if p <0.05 in univariate analysis - shrinkage: NR | |
| Model performance | - calibration: NR, - discrimination: AUC - classification measures reported, with fixed FPRs | - calibration: Hosmer-Lemeshow - discrimination: AUC - dlassification measures for different screening cut-offs | | - calibration: NR - discrimination: NR - classification measures for different strategies | | - calibration: NR - discrimination: NR - classification measures reported | | - calibration: NR - discrimination AUC - classification measures reported | |
| Model evaluation | - NR | - internal validation: random split sample, no adjustment or update | | - NR | | - NR | | - NR | |
| Results | - model with odds ratios reported - intercept: NR - separate model for women without hx of GDM | - model with odds ratios presented - intercept: NR - simple clinical scoring system | | - RECPAM classes with odds ratios presented - intercept: NR | | - decision tree - univariate odds ratios | | - full regression equation presented - classification measures for different strategies reported | |
| Interpretation and discussion | - screening by prediction model superior to risk factor screening - validation in prospective studies | - selective screening could spare numerous OGTTs | | - screening approach reduces undetected GDM cases | | - early identification of high risk for GDM is possible - larger studies to early detection and screening for GDM | | - a composite model may reduce the need for universal screening | |
|  | **9. Savvidou 2010** | **10. Shirazian 2009** | | **11. Syngelaki 2011** | | **12. Teede 2011** | | **13. Tran 2013** | |
| Objective | Predictive ability of routinely booking variables for GDM [D] | Influence of risk factors on incidence of GDM [D] | | Use BMI to calculate patient specific risk for GDM [D] | | Develop and validate a scoring system for predicting GDM [D] | | Evaluate the performance of prognostic models for GDM [D] | |
| Source of data | Nested case-control study | Prospective cohort | | Prospective cohort | | Retrospective cohort | | Prospective cross-sectional study | |
| Participants | - 372 participants, dates NR, single centre, UK - recruitment: 11^+0^ – 13^+6^ wks - exclusion: hx of DM, multiple gest. - baseline characteristics presented | - 971 participants, 2005 - 2008, multicentre, Iran - recruitment: at booking - exclusion: >28 wks at first visit, hx of DM, comorbid conditions - baseline characteristics presented | | - 41.577 participants, dates NR, multicentre, UK - recruitment at 11^+0^-13^+6^ wks - exclusion: IUI, aneuploidies or major defects, hx of DM, TOP, miscarriage or delivery <30 wks - baseline characteristics described | | - 4276 participants, 2007 - 2008, single centre, Australia - recruitment: 12-15 wks - exclusion: multiple gest. - baseline characteristics presented | | - 2772 participants, 2010-2011, single centre, Vietnam - recruitment: 24-32 wks - exclusion: age <18 yrs, hx of DM, multiple gest., uncertain GA - baseline characteristics presented | |
| Outcomes to be predicted | - GDM: WHO - 2-step: risk factor, 24-28 wks - outcome assessment not blinded | - GDM: ADA - 1-step, 24 -28 wks - outcome assessment not blinded | | - GDM: WHO II - 2-step: glucose (6.7), 24-28 wks - outcome assessment not blinded | | - GDM: ADIPS - 2-step: 50g GCT (8.0), 24-28 wks - outcome assessment not blinded | | - GDM: ADA, IADPSG, WHO, ADIPS - 1-step, 24-32 wks - outcome assessment blinding NR | |
| Candidate predictors | - questionnaire on demographics, patient history; physical examination - 5 continuous, 6 dichotomous variables - predictor assessment at booking | - questionnaire on demographics, patient history; physical examination - 3 continuous, 12 dichotomous variables - predictor assessment at booking | | - questionnaire on demographics, patient history; physical examination - 2 continuous, 1 categorical, 6 dichotomous variables - predictor assessment at booking | | - demographics, patient history, physical examination recorded - 2 continuous; 5 dichotomous variables - predictor assessment at booking | | - interview on demographics, patient history; physical examination - 3 continuous, 1 categorical, 8 dichotomous variables - predictor assessment at testing | |
| Sample size | - 124 GDM cases - EPV 11.3 | - 68 GDM cases (7.4%) - EPV 4.5 | | - number of events: NR - EPV NR | | - D: 2880, 250 GDM cases (8.9%) - V: 1396, 106 GDM cases (7.6%) - EPV 35.7 | | - 164-674 GDM cases (5.9 – 24.3%) - EPV: 14 | |
| Missing data | - missing data: NR | - excluded if missing outcome (4.8%) | | - complete case analysis - 6.1% with missing outcome | | - A: missing data: NR - B: 167 excluded for missing predictors (11.9%) | | - complete case analysis - excluded if missing data (6.0%) | |
| Model development | - multivariable logistic regression - predictor selection: forward stepwise, backward and bootstrap selection - shrinkage: NR | - multivariable logistic regression - predictor selection: backward, criteria: NR - shrinkage: NR | | - multivariable logistic regression - predictor selection: all candidate, criteria: NR - shrinkage: NR | | - multivariable logistic regression - predictor selection: if p<0.05 in univariate analysis - shrinkage: NR | | - predictor selection by Bayesian model averaging, criteria: NR | |
| Model performance | - calibration: NR - discrimination: AUC | - calibration: NR - discrimination: NR - classification measures reported | | - calibration: NR - discrimination: NR - classification measures: NR | | - calibration: NR - discrimination: AUC - classification measures for each score | | - calibration: NR - discrimination: AUC - classification measures reported | |
| Model evaluation | - NR | - NR | | - NR | | - split sample by date - no adjustment or update | | - NR | |
| Results | - full regression model for model including biomarkers - separate model for women without hx of GDM | - regression model with odds ratios - intercept: NR - clinical scoring system | | - regression model with odds ratios - intercept: NR | | - regression model with odds ratios - intercept: NR - clinical scoring system | | - RR for each predictor in model - nomogram for ADA model - classification measures per diagnostic criteria | |
| Interpretation and discussion | - very good prediction of GDM with clinical characteristics - validation of prediction model | - selective screening will not miss a substantial number of GDM cases - larger studies are needed | | - model can form the basis for patient-specific risks for pregnancy complications | | - scoring tool is easy to develop if database is available - prediction tool enables prevention | | - a simple prognostic model can be used in low- and middle-income settings | |
|  | **14. Van Leeuwen 2010** | | **15. Lovati 2013** | | **16. Theriault 2013** | | **17. Van Leeuwen 2009** | |  |
| Objective | Development of prediction model for GDM [D] | | Predictive value of PAPP-A and standard risk factors for GDM [V] | | Evaluate the performance of prediction models for GDM [V] | | Validate scoring system of Naylor 1997 in an external population [V] | |  |
| Source of data | Prospective cohort | | Case-control study | | Prospective cohort | | Prospective cohort | |  |
| Participants | - 995 participants, dates NR, single centre, Netherlands - recruitment: <20 wks - exclusion: hx of DM, multiple gest. - baseline characteristics: NR | | - 673 participants, 2009-2011, single centre, Italy - recruitment: 24-28 wks - inclusion: primiparous - exclusion: hx of DM, aneuploidy, multiple gest., hx of hypertension - baseline characteristics presented | | - 7208 participants, 2005 - 2010, multi centre, Canada - recruitment: < 20wks - exclusion: age < 18 yrs, renal or hepatic disease, multiple gest., hx of DM - baseline characteristics presented | | - 1.301 consecutive women, single centre, Netherlands - recruitment: <24 wks - exclusion: pre-existing DM, multiple gestation - baseline characteristics presented | |  |
| Outcomes to be predicted | - GDM: WHO - 2-step: 50g GCT (7.8) + random glucose (6.8), 24-28 wks - outcome assessment not blinded | | - GDM: Carpenter&Coustan till 2010, IADPSG from 2010 - 1-step, 24-28 wks - outcome assessment not blinded | | - GDM: CDA - 2-step: 50g GCT (7.8), wks NR outcome assessment not blinded | | - GDM: WHO - 2-step: 50g GCT (7.8) + random glucose (6.8), 24-28 wks - outcome assessment not blinded | |  |
| Candidate predictors | - demographics, patient history; physical examination recorded - 2 continuous, 2 categorical, 1 dichotomized, 2 dichotomous variables - predictor assessment at booking | | - V: 1 model (Teede ’11) - questionnaire on demographics, patient history; physical examination - predictor assessment at booking | | - V: 4 models: (Caliskan ’04, Naylor ’97, Teede ’11, v Leeuwen ’10) - questionnaire on demographics, patient history; physical examination - predictor assessment 24-28 wks | | - V: 1 model (Naylor ’97) - demographics, patient history; physical examination at booking - predictor assessment at booking | |  |
| Sample size | - 24 GDM cases (2.4%) - EPV: 3 | | - 307 GDM cases | | - 381 GDM cases (5.3%) | | - 47 GDM cases (3.7%) | |  |
| Missing data | - missing data: NR - multiple imputation | | - missing data: NR | | - complete case analysis - missing outcome (5.0%) | | - missing predictor (2.7%) - multiple imputation | |  |
| Model development | - multivariable logistic regression - predictor selection: backward (P <0.30 in; P > 0.20 out) - shrinkage: uniform | | - n.a. | | - n.a. | | - n.a. | |  |
| Model performance | - calibration: Hosmer-Lemeshow, plot on request - discrimination: AUC - classification measures reported | | - calibration: NR - discrimination: AUC - classification measures reported | | - calibration: NR - discrimination: AUC, Youden index - classification measures reported | | - calibration: _X_^2^ goodness of fit test - discrimination: AUC - classification measures reported | |  |
| Model evaluation | - NR | | - no recalibration - no adjustment or update | | - no recalibration - no adjustment or update | | - no recalibration - no adjustment or update | |  |
| Results | - full regression model - nomogram - classification measures for different cut-off points | | - model with odds ratios reported - risk factor scoring presented | | - model performance for subgroups: insulin dependent GDM & GDM dietary only | | - observed prevalence compared with original study | |  |
| Interpretation and discussion | - the prediction model is accurate for clinical use - external validation is required | | - less significant than validation study - studies for further validation are needed | | - performance similar to original studies, promising for early prediction of GDM insulin - improvement by biomarkers might be possible | | - risk score by Naylor 1997 is unsatisfactory - explore new prediction models | |  |

Abbreviations: *D*, development study; *V*, validation study; *wks*, weeks of gestation; *hx,* history; *DM*, diabetes mellitus; *gest.*, gestation; *NR*, not reported; *GDM*, gestational diabetes; *USA*, United States of America; *GCT*, glucose challenge test; *IADPSG*, the International Association of the Diabetes and Pregnancy Study Groups; *EPV*, events-per-variable; *FPR*, false-positive rate; *AUC*, area under the curve; *PlGF*, placental growth factor; *UK*, United Kingdom; *yrs*, years; *WHO*, World Health Organization; *ADA*, American Diabetes Association; *RECPAM*, Recursive Partitioning and Amalgamation; *IUI*, intra-uterine insemination; *TOP*, termination of pregnancy; *GA*, gestational age; *ADIPS*, Australasian Diabetes in Pregnancy Society; *RR*, relative risk; *PAPP-A*, pregnancy-associated plasma protein-A; *CDA*, Canadian Diabetes Association

^a^ Glucose values are given in mmol/L.
